# Risk factors for clopidogrel resistance in patients with ischemic cerebral infarction and the correlation with *ABCB1* gene rs1045642 polymorphism

**DOI:** 10.3892/etm.2014.2058

**Published:** 2014-11-10

**Authors:** JUN-FENG SU, XIAO-HUI HU, CHENG-YAN LI

**Affiliations:** 1 Department of Neurology, Renmin Hospital of Wuhan University, Wuhan, Hubei 430060, P.R. China; 2 Department of Neurology, Jingzhou Central Hospital, Tongji Medical College, Huazhong University of Science and Technology, Jingzhou, Hubei 434020, P.R. China

**Keywords:** ischemic stroke, clopidogrel resistance, *ABCB1* gene, polymorphisms

## Abstract

The aim of the present study was to examine clopidogrel resistance (CR) in patients with ischemic cerebral infarction and its potential association with a single nucleotide polymorphism (SNP; rs1045642) in the *ABCB1* gene. Patients with ischemic cerebral infarction received clopidogrel (75 mg/day) for 7 days and were then subjected to a turbidimetric assay to determine platelet aggregation. Patients were then divided into a CR group and a clopidogrel-sensitive (CS) group. Demographic and clinical data between the two groups were compared. Multivariate logistic regression analysis was performed to determine independent risk factors of CR. PCR products were sequenced to assess *ABCB1* rs1045642 SNP genotype and allele frequencies in each group. In total, 303 patients were enrolled in the study; this included 51 CR cases (16.83%) and 252 CS cases (83.17%). Several parameters, including hypertension, diabetes, calcium channel blocker (CCB), β-receptor blocking agent and proton pump inhibitor use, and creatinine, fasting blood glucose, homocysteine (HCY), high-sensitivity C-reactive protein (hs-CRP) and triglyceride levels were significantly higher in the CR group than in the CS group. Diabetes, hs-CRP-increased use of CCBs, and use of β-blockers were found to be independent risk factors for CR. However, *ABCB1* gene rs1045642 polymorphism was not found to be an independent risk factor for CR. In conclusion, CR in ischemic stroke patients is associated with several independent risk factors, including diabetes, hs-CRP-increased use of CCBs, and use of β-blockers. However, *ABCB1* gene rs1045642 polymorphism has no correlation with CR.

## Introduction

Clopidogrel is an anti-platelet agent used to prevent blood clots. It selectively and irreversibly inhibits the P2Y12 class of adenosine diphosphate (ADP) receptors on platelets to prevent aggregation. Although a number of studies have confirmed the clinical efficacy of clopidogrel as an anti-thrombotic agent, its efficiency in preventing platelet aggregation is not uniform in all patients. Between 4 and 44% of patients receiving this drug display a poor response (1,2). The patients that fail to respond are referred to as clopidogrel resistant (3).

While the exact reason for clopidogrel resistance (CR) is not clear (4), the response to the drug is influenced by genetics and by clinical and pathophysiological factors (5,6). Clopidogrel is a pro-drug. That is, before it can exert its anti-platelet activity, it must be metabolized by the liver into its active form; it is only this active metabolite of clopidogrel that is able to bind the ADP receptors on platelets to prevent aggregation (7). Intestinal epithelial cells expressing P-glycoprotein (P-gp) can influence the absorption of clopidogrel from the digestive tract into the blood, thus affecting the efficacy of the drug (8,9). ABCB1 is expressed in P-gp epithelial cells (10), and the distribution of P-gp differs among individuals; this may explain the differential response of patients to clopidogrel (10). Recent studies have shown that single nucleotide polymorphisms (SNPs) within the *ABCB1* gene affect its degree of transcription and translation; subsequently, clopidogrel response may also be affected. Simon *et al* reported that the serum concentration of the active metabolite of clopidogrel is reduced in individuals with the *ABCB1* gene 3435C>T SNP (7). Another study reported that in patients treated with clopidogrel, *ABCB1* 3435C>T polymorphism, T allele carriers and major adverse cardiovascular events (MACE) are all closely associated with risk (11). These earlier studies focused on coronary heart disease patients, whose disease pathogenesis differs from those with ischemic stroke. Thus, in the present study, the risk factors of Chinese patients with ischemic cerebral infarction were explored and the correlation between *ABCB1* gene polymorphisms and CR was examined. The data obtained may help to improve individualized antiplatelet treatment options for the ischemic stroke population and reduce adverse side-effects.

## Materials and methods

### Subjects

Patients with ischemic cerebral infarction who were seen at Jingzhou Central Hospital of Tongji Medical College, Huazhong University of Science and Technology (Jinzhou, China) between June 2013 and December 2013 were enrolled in the present study if they met the following inclusion criteria: i) older than 18 years; ii) present or past diagnosis of ischemic stroke [according to the 2010 Chinese Guidelines for the Management of Ischemic Stroke (12), with clinical symptoms and computed tomography (CT) or magnetic resonance imaging (MRI) verification]; iii) Trial of Org 10172 in Acute Stroke Treatment (TOAST) classification (13) of large artery atherosclerosis or small arteries with non-cardiac ischemic stroke. Patients who met the following exclusion criteria were not enrolled: i) 10 days of treatment with clopidogrel, ticlopidine, dipyridamole or other anti-platelet drugs, or treatment with other non-steroidal drugs; ii) 24 h of treatment with standard or low-molecular-weight heparin; iii) major surgery within the preceding week; iv) occurrence of any bleeding disorders or a family history of blood diseases; v) kidney or heart dysfunction or other serious systemic diseases; vi) concurrent malignancy or ongoing anti-tumor therapy; vii) platelet count <150×10^12^/l or >450×10^12^/l, and hemoglobin <8 g/l. The study and all procedures were approved by the ethics committee of Tongji Medical College, Huazhong University of Science and Technology, and all patients enrolled in the provided signed informed consent.

### Information collection

Information was obtained from all patients on their general condition, medical history (hypertension, diabetes or coronary heart disease), personal history (smoking and drinking), laboratory tests, and use of concomitant medications, namely calcium channel blockers (CCBs), angiotensin-converting enzyme inhibitors, β-blockers, lipid-lowering drugs and proton pump inhibitors.

### Determination of platelet aggregation

The turbidimetric method using optical density (1ight transmission aggregation, LTA) was performed to assess ADP (20 μmol/l)-induced platelet aggregation. Prior to collection of the specimens, the patients had received clopidogrel (75 mg) daily for one week. Blood (2ml) was extracted from the cubital vein of each patient in the morning on an empty stomach; two tubes of blood were filled and gently inverted to prevent clotting. Then, to prepare platelet-rich plasma (PRP), the samples were first spun at 700 × g for 10 min, and the sample quality, including severe hemolysis and severe lipemia, was determined. The supernatant containing PRP was removed, placed in a separate tube, and stored at room temperature until use. PRP was separated from platelet-poor plasma (PPP) by centrifugation at 2,200 × g for 10 min. For test sampling, 300 μl of each sample (PRP and PPP) was drawn, and platelet aggregation was determined using an SC-2000 platelet testing apparatus (Beijing Saikexide Technology Development Co., Ltd., Beijing, China); platelet aggregation was induced by treatment with ADP (20 μmol/l). A platelet aggregation rate ≥70% was defined as CR, and a platelet aggregation rate <70% was defined as clopidogrel-sensitive (CS). All experiments were performed within 3 h.

### Genomic DNA extraction and genotyping

Genomic DNA was isolated from 1 ml peripheral blood using a TIANamp Blood DNA kit [DP318; Tiangen Technology (Beijing) Co., Ltd., Beijing, China]. Samples were stored at −20°C until use. Primers used for genotyping were as follows: (rs1045642-F: 5-′GAATGTTCAGTGGCTCCGA-3′ and rs1045642-R: 5′-CTCCCAGGCTGTTTATTTGA-3′). PCR amplification (BTC-1000; Bio Basic, Markham, Canada) was performed as follows: 50 μl reaction volume containing template DNA 1 μl, upstream and downstream primers 1 μl, dNTP 10 mM 1 μl, Taq Buffer 5 μl, 25 mM MgCl_2_, 5 μl Taq enzyme (5 U/μl) 0.5 μl and water 35.5 μl. PCR cycling conditions consisted of 95°C denaturation for 3 min, 35 cycles of 94°C denaturation for 30 sec, 56°C annealing for 35 sec, and 72°C extension for 40 sec, and finally one cycle of 72°C repair extension for 5 min. PCR products were purified (SK1141 kit; Sangon Biotech, Shanghai, China), measured using a 3730 DNA sequence analyzer (Applied Biosystems, Foster City, CA, USA), and sequenced.

### Statistical analysis

SPSS software package, version 17.0 (SPSS, Inc., Chicago, IL, USA) was used to conduct the statistical analysis. Quantitative data are presented as the mean ± standard deviation or median (quartiles). According to the nature of the statistical data, classification was performed by χ^2^ test, corrected χ^2^ test or Fisher’s exact test. Normality and homogeneity of variance were determined by t-test between two independent samples; non-parametric Wilcoxon test was used for this type of analysis. P<0.05 was considered to indicate a statistically significant difference. Univariate analysis (P<0.05) and significant professional binary logistic regression analysis were performed to assess independent risk factors.

## Results

### CR and ischemic stroke risk factors, correlation of laboratory tests and concomitant medication

A total of 303 cases (93 females and 210 males) met all requirements and were included in the study. Cases ranged in age from 29 to 82 years, with a mean age of 63.65±9.60 years. There were 51 cases (16.83%) of CR, and 252 cases (83.17%) were categorized into the CS group.

Univariate analysis demonstrated that several risk factors associated with ischemic stroke, including hypertension and diabetes, correlated with CR (Table I). Similarly, treatment with CCBs, β-receptor blocking agents and proton pump inhibitors was also associated with CR. Laboratory tests for creatinine (Cr), fasting blood glucose (FBG), homocysteine (HCY), high-sensitivity C-reactive protein (hs-CRP), and triglycerides also confirmed that each of these factors positively correlated with CR (P<0.05; Table I). CR occurred in 20% of patients with hypertension; by contrast, only 7.69% of individuals without hypertension experienced resistance to the drug. CR was also more common in diabetic patients (54.55%) than in non-diabetic patients (12.22%). It was also observed that CR was associated with the administration of various agents, including CCBs (χ^2^=10.566, P<0.01), β-blockers (χ^2^=14.052, P<0.001) and proton pump inhibitors (χ^2^=12.767, P<0.001) and with several laboratory tests, including measures of Cr (Z=2.286, P<0.05), FBG (Z=3.375, P<0.01), HCY (Z=2.31, P<0.05), hs-CRP (Z=6.582, P<0.01) and triglycerides (Z=2.35, P<0.05). No significant association was identified between response to clopidogrel and gender, coronary heart disease or smoking.

Univariate analysis identified several statistically significant independent variables, including hypertension, diabetes, CCBs, β-receptor blocking agents, proton pump inhibitors, Cr, FBG, HCY, hs-CRP, and triglycerides which were associated with CR as dependent variables. Controlling for gender, smoking and other factors, binary logistic regression analysis identified several additional independent risk factors associated with CR, including CCBs, β-blockers, hs-CRP and diabetes (Table II).

### Correlation among ischemic stroke, ABCB1 gene rs1045642 polymorphism and CR

Polymorphisms of the *ABCB1* gene (rs1045642) are shown in Table III. The two polymorphisms were found to be in Hardy-Weinberg equilibrium. Univariate analysis revealed that there was a significant difference between the CR and CS groups (P=0.006, χ^2^=10.383). Moreover, there were different associations between each of the genotypes and response to clopidogrel. For example, there was no significant difference in the C, T allele frequency between the CR and CS groups (P=0.223, χ^2^=1.488). Controlling for gender, smoking, and other factors, binary logistic regression analysis was performed and it was found that none of the three genotypes were independent risk factors for CR (P=0.0192, P=0.070 and P=0.118).

## Discussion

The majority of previous studies examining CR have focused on individuals with cardiovascular disease. By contrast, less is known about the response to clopidogrel in patients suffering from ischemic stroke. In the present study, a 16.83% incidence of CR was observed, which is consistent with the rates published in the literature (4–44%) (1,2). However, there are differences between cardiovascular and cerebrovascular conditions that is it necessary to address.

The exact mechanisms responsible for CR are unknown. However, several studies have shown that self-compliance, inadequate dosing, absorption or metabolic dysfunction, drug interactions, diabetes, vascular factors, and genetic factors may all influence the response to the drug (14–17). Other studies have shown a decreased response to clopidogrel in patients with elevated levels of glycated hemoglobin and C-peptide (18,19). Diabetes has also been described as an independent risk factor for CR (20). In the present study, it was found that the long-term use of CCBs, hs-CRP and β-blockers is associated with CR. While the association between CCBs and clopidogrel has been previously reported (21), to the best of our knowledge, this is the first time any correlations between β-blockers, hs-CRP and clopidogrel response have been described. These findings suggest that patients with these factors should be given more attention, with regular testing for platelet aggregation. It is important to identify patients with CR, as they may benefit from additional anti-platelet drugs to prevent recurrent stroke.

Recent studies have focused on the identification of potential correlations between CR and genetic polymorphisms (22,23). Clopidogrel is a pro-drug that must be metabolized to its active form. Two genes important for drug metabolism are *ABCB1* and *CYP2C19*. *CYP2C19* has been previously linked to CR (24,25). Associations between *ABCB1* and CR are not as well established and studies have reported conflicting results. One study found that patients with the *ABCB1* TT genotype at the rs1045642 locus had a higher risk of recurrent ischemic events following clopidogrel treatment; those with the wild-type CC genotype did not experience this increased rate of recurrence (11). In contrast to these findings, another study reported that individuals with the wild-type CC genotype had a higher risk of recurrent ischemic events following clopidogrel treatment (26). Importantly, these earlier studies focused on coronary heart disease patients and not on stroke sufferers. In the present study, the potential correlation between *ABCB1* genotype and ischemic stroke recurrence was examined in patients treated with clopidogrel. Notably, no significant correlation was identified among these variables. This could be due to confounding factors. Additionally, a single polymorphic locus may not be directly responsible for the effect; instead, an analysis of multiple polymorphic loci may require consideration.

In conclusion, the data presented in the present study suggest that several patient characteristics should be considered prior to the administration of clopidogrel. This would help distinguish patients that are likely to respond to the drug from those that would likely be resistant. While this study is promising, it is partly limited by its small sample size. It should be followed up by another multi-center clinical trial with a greater number of patients in order to confirm and verify these findings.

## Figures and Tables

**Table I:** Comparison of clinical data between the clopidogrel resistant and sensitive groups.

		Clopidogrel			
				
Variable	Class	Sensitive (n=252)	Resistant (n=51)	Statistics^a^	P-value
Gender	Female	72 (77.42)	21 (22.58)	3.168	0.075
	Male	180 (85.71)	30 (14.29)		
Hypertension	No	72 (92.31)	6 (7.69)	6.268	0.012
	Yes	180 (80.00)	45 (20.00)		
Coronary heart disease	No	192 (85.33)	33 (14.67)	2.927	0.087
	Yes	60 (76.92)	18 (23.08)		
Diabetes	No	237 (87.78)	33 (12.22)	37.628	<0.001
	Yes	15 (45.45)	18 (54.55)		
Smoking	No	153 (80.95)	36 (19.05)	1.762	0.184
	Yes	99 (86.84)	15 (13.16)		
Drinking	No	198 (84.62)	36 (15.38)	1.537	0.215
	Yes	54 (78.26)	15 (21.74)		
CCB	No	165 (88.71)	21 (11.29)	10.566	0.001
	Yes	87 (74.36)	30 (25.64)		
ACEI	No	216 (84.71)	39 (15.29)	2.718	0.099
	Yes	36 (75.00)	12 (25.00)		
β-blocker	No	237 (85.87)	39 (14.13)	14.052	<0.001
	Yes	15 (55.56)	12 (44.44)		
Statin	No	15 (100.00)	0 (0.00)	2.054	0.152
	Yes	237 (82.29)	51 (17.71)		
Proton pump inhibitor	No	234 (85.71)	39 (14.29)	12.767	<0.001
	Yes	18 (60.00)	12 (40.00)		
Age (years)		63 (56.5,71)	67 (62,69)	1.941	0.052
BUN (mmol/l)		5.03 (4.05,6.23)	4.2 (3.85,5.82)	−1.561	0.119
Cr (umol/l)		72.95 (59.15,88.1)	79.1 (60.9,111.5)	2.286	0.022
UA (umol/l)		319.9 (255.15,391.85)	367.3 (273.5,429.1)	1.845	0.065
FBG (mmol/l)		5.57 (5.11,6.57)	6.11 (5.49,8.02)	3.375	0.001
HCY (umol/l)		15.92 (13.44,21.28)	19.46 (15.46,22.65)	2.31	0.021
hs-CRP (mg/l)		0.95 (0.5,2.65)	4.21 (2.21,11.97)	6.582	<0.001
Cholesterol (mmol/l)		4.72 (3.87,5.59)	4.96 (3.82,5.34)	−0.449	0.654
Triglycerides (mmol/l)		1.31 (0.95,2.05)	1.39 (1.23,2.83)	2.35	0.019
HDL (mmol/l)		1.04 (0.91,1.25)	1.08 (0.89,1.34)	0.614	0.539
LDL (mmol/l)		2.68 (1.98,3.39)	2.7 (2,2.92)	−1.679	0.093
Platelet count (×10^9^)		157.5 (135.15,193.7)	173 (127,206.3)	0.299	0.765

CCB, calcium channel blocker; ACEI, angiotensin-converting enzyme inhibitor; BUN, blood urea nitrogen; Cr, creatinine; UA, uric acid; FBG, fasting blood glucose; HCY, homocysteine; hs-CRP, high-sensitivity C-reactive protein; HDL, high-density lipoprotein; LDL, low-density lipoprotein.

a Some of the statistics are χ^2^ values and some are Z values.

**Table II:** Logistic regression analysis.

							OR, 95% CI	
							
Variable	B	SE	Walds	df	P-value	OR	Limit	Cap
CCB	1.137	0.359	10.046	1	0.002	3.117	1.543	6.296
β-blockers	1.406	0.497	8.022	1	0.005	4.081	1.542	10.802
hs-CRP	.056	0.018	9.463	1	0.002	1.058	1.021	1.097
Diabetes	2.149	0.442	23.597	1	0.000	8.575	3.603	20.407
Constant	−3.039	0.333	83.446	1	0.000	0.048		

CCB, calcium channel blocker.; hs-CRP, high-sensitivity C-reactive protein; SE, standard error; df, degree of freedom; OR, odds ratio; CI, confidence interval.

**Table III:** *ABCB1* rs1045642 genotype distribution and allele frequencies.

Genotype and allele	Clopidogrel resistance (n=51)	Clopidogrel sensitive (n=252)	χ^2^ value	P-value
CC, n (%)	27 (23.7)	87 (76.3)	10.383	0.006
TT, n (%)	6 (28.6)	15 (71.4)		
CT, n (%)	18 (10.7)	150 (89.3)		
C, n (%)	72 (18.2)	324 (81.8)	1.488	0.223
T, n (%)	30 (14.3)	180 (85.7)		
